# Applications of machine learning in metabolomics: Disease modeling and classification

**DOI:** 10.3389/fgene.2022.1017340

**Published:** 2022-11-24

**Authors:** Aya Galal, Marwa Talal, Ahmed Moustafa

**Affiliations:** ^1^ Systems Genomics Laboratory, American University in Cairo, New Cairo, Egypt; ^2^ Institute of Global Health and Human Ecology, American University in Cairo, New Cairo, Egypt; ^3^ Biotechnology Graduate Program, American University in Cairo, New Cairo, Egypt; ^4^ Department of Biology, American University in Cairo, New Cairo, Egypt

**Keywords:** metabolomics, machine learning, metabolic disorders, biomarkers, deep learning

## Abstract

Metabolomics research has recently gained popularity because it enables the study of biological traits at the biochemical level and, as a result, can directly reveal what occurs in a cell or a tissue based on health or disease status, complementing other omics such as genomics and transcriptomics. Like other high-throughput biological experiments, metabolomics produces vast volumes of complex data. The application of machine learning (ML) to analyze data, recognize patterns, and build models is expanding across multiple fields. In the same way, ML methods are utilized for the classification, regression, or clustering of highly complex metabolomic data. This review discusses how disease modeling and diagnosis can be enhanced via deep and comprehensive metabolomic profiling using ML. We discuss the general layout of a metabolic workflow and the fundamental ML techniques used to analyze metabolomic data, including support vector machines (SVM), decision trees, random forests (RF), neural networks (NN), and deep learning (DL). Finally, we present the advantages and disadvantages of various ML methods and provide suggestions for different metabolic data analysis scenarios.

## Introduction

Metabolomics is the study of small metabolites or chemical processes involving small substrates in tissues or organisms. The metabolome is the representation of all metabolites in any biological cell, tissue, or organ and their subsequent cellular products. It provides a snapshot of the physiology of the cell under investigation and can be used to study biological information on the biochemical level. This provides an avenue of study that leads to understanding the biological phenotype, which can be used in the context of health and disease (Gowda et al., 2008). Roger Williams introduced the concept of a metabolic profile in the late 1940s (Gates and Sweeley 1978). He used paper chromatography to suggest that schizophrenia presents characteristic metabolic patterns in urine and saliva. Only with the technological advancements of the 1970s and with the introduction of gas chromatography and mass spectrometry was the term “metabolic profile” introduced (Griffiths and Wang 2009). The first comprehensive metabolomic tandem mass spectrometry database, Metabolite and Chemical Entity Database (METLIN), was developed in 2005 by the Scripps Research Institute (Smith et al., 2005; Guijas et al., 2018). In 2007, “The Human Metabolome Project,” led by David S. Wishart, established the first draft of a database with ∼2,500 metabolites, ∼1,200 drugs, and ∼3,500 food components (David S. Wishart et al., 2007; Wishart et al.,. 2009). Now, techniques such as mass spectrometry and gas chromatography have advanced so that they can detect thousands of independent features in a single specimen, making identifying metabolites associated with a disease or trait an increasingly difficult computational challenge. The field of metabolomics has enabled a comprehensive assessment of biological specimens and their associated compounds. This improved understanding of the biological system at the molecular level is crucial in aiding disease diagnosis and therapeutic development (Gowda et al., 2008). Within the omics field, metabolomics represents the underlying layer that reflects all information expressed and modulated by the upstream genetic regulation and processing layers. It is the closest link to the phenotype. It is at the forefront of personalized health, in terms of diagnosis and therapy, through its direct applicability to the area of biomarker discovery (Shah, Sureshkumar, and Shewade 2015; Aderemi et al., 2021). Biological systems are complex and often require the integration of several layers of omic data to decipher. Metabolomics is a potential solution for this, as it represents the product of the interaction between the various omic layers (Hasin, Seldin, and Lusis 2017; Misra et al., 2018).

Metabolic disorders are biochemical aberrations that can be detected through screening techniques or biomarker identification. However, biomarker identification requires extensive prior knowledge and numerous disease models for a single biomarker to be successfully linked to a disease. Metabolomics and other “omics” molecular profiling techniques provide essential tools for discovering new disease risk factors and biomarkers (Smith et al., 2005; Gowda et al., 2008) without the typical hurdles of time and money. The most studied metabolic disorders include diabetes mellitus (DM) (Friedrich 2012; Guasch-Ferré et al., 2016; Ahola-Olli et al., 2019; Sun et al., 2019; Hou, Wang, and Pan 2021), cardiovascular disease (CVD) (Müller et al., 2021; Iida, Harada, and Takebayashi 2019; Streese et al., 2021; McGranaghan et al., 2020; Cavus et al., 2019; Ruiz-Canela et al., 2017), and cancers (Gowda et al., 2008; Raffone et al., 2020; Yang et al., 2020; Schmidt et al., 2021).

**FIGURE 1:**
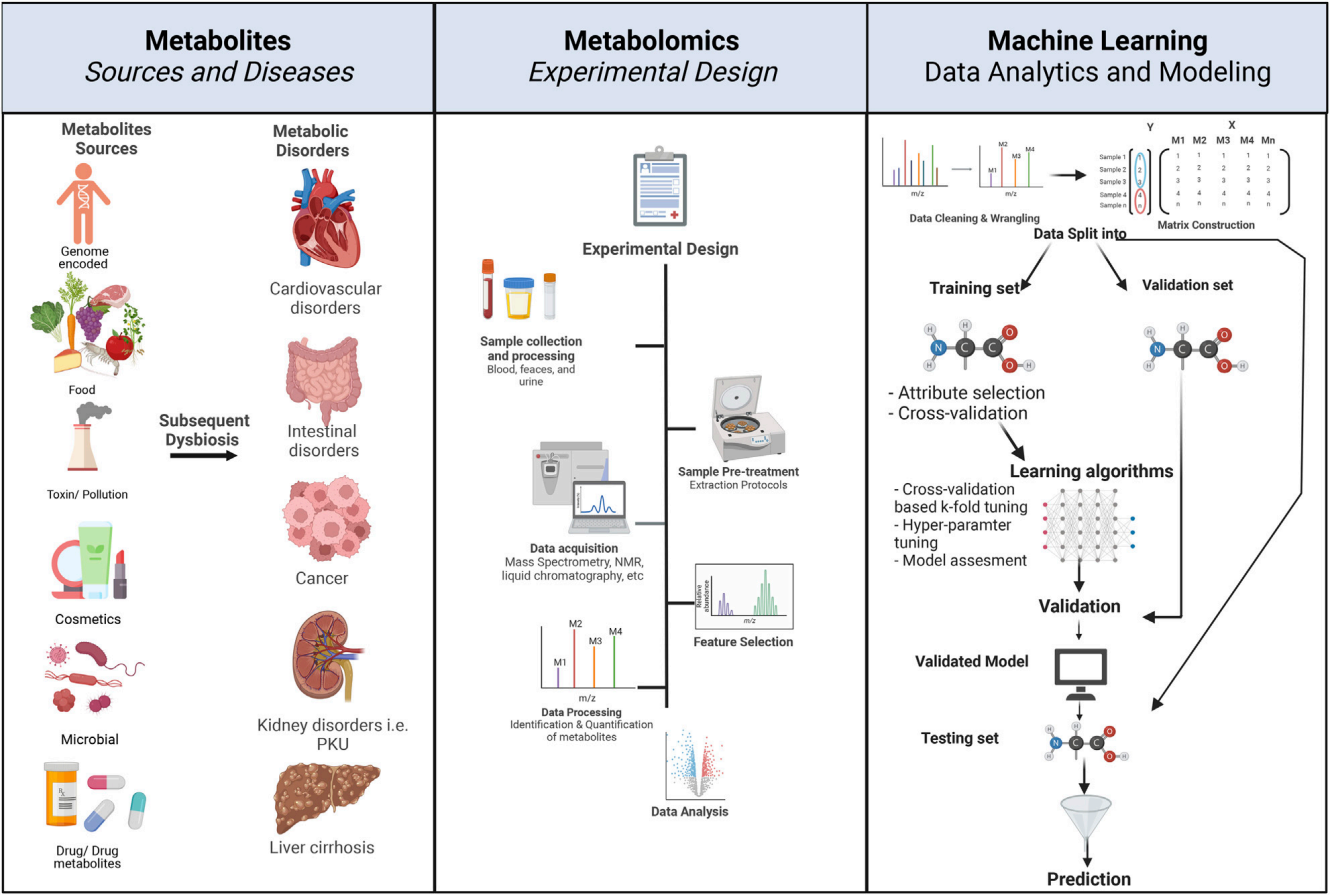
Principles of metabolomics experimental design and associated ML workflow. The left panel describes the various sources of metabolites. Metabolite exposure can be through endogenous and exogenous means, e.g., human-encoded, microbiome-encoded, food, drugs, and toxins. Metabolic dysbiosis can be associated with metabolic disorders, e.g., cancer, cardiovascular disease, intestinal disorders, and diabetes. The center panel describes the typical flow and design of a metabolomic experiment, starting with the 1) study design where disease and control groups are determined, 2) followed by sample selection, e.g., urine, stool, blood, and serum, 3) collected samples undergo pre-treatment and processing according to experimental design, 4) data acquisition, e.g., through mass spectrometry or NMR, 5) feature selection involves the identification of desired metabolite features that will undergo subsequent, 6) data processing through the quantification of metabolites, and finally, 7) data analysis depends on the study design. The right panel describes the concepts of ML workflow and prediction, starting with 1) data wrangling and cleaning, 2) matrix construction, where data from each metabolite is placed in a matrix in reference to the conditions, i.e., disease (marked in red), control (marked in blue), 3) data are then divided into testing, validation and training datasets, 4) ML algorithm is applied, and 5) cross-validation, and testing of the predictive power of the algorithm on a test dataset. Created with BioRender.com.

For the purposes of this review, the main metabolomic experimental workflow can be divided into four main parts: 1) sample retrieval and preparation, 2) separation and detection of metabolites, 3) data processing, including data mining and extraction, and 4) data analysis (Figure 1, Middle Panel). Sample retrieval and preparation depend on the type of material to collect. Metabolites can be measured from a variety of different biological samples, e.g., tissue, biofluids, and cell culture. Depending on the disease or trait under investigation, the choice of specimen differs, as do the steps required to prepare the sample for the corresponding experiment. For example, tissue specimens should be immediately quenched with liquid nitrogen after harvesting to arrest the metabolism. Numerous sample preparation protocols entailing the details of metabolite extraction, enrichment, and depletion of proteins have been developed (Dettmer, Aronov, and Hammock 2007; D. S. Wishart 2005; Want et al., 2007). Separation and detection of metabolites can be achieved by two main protocols: nuclear magnetic resonance (NMR) and mass spectrometry and their assorted subtypes (Gowda et al., 2008). Both techniques are capable of high-throughput measurements of a large number of metabolites.

Metabolomics studies can be subclassified into three major approaches: targeted analysis (Shulaev 2006; Griffiths and Wang 2009; Mookherjee et al., 2020), metabolite profiling, i.e., untargeted analysis (Fiehn 2002; Halket et al., 2005), and metabolic fingerprinting, which is also known as exometabolomics and focuses on extracellular metabolites while utilizing analytical profiling approaches (Allen et al., 2003; Mapelli, Olsson, and Nielsen 2008; Silva and Northen 2015; Thomas et al., 2021). Targeted approaches are limited to a set of predetermined metabolites of interest for identifying and quantifying these specific metabolites. Untargeted approaches are conducted to identify a comprehensive metabolic profile in a specimen. The choice of metabolomics workflow and the associated downstream steps depends on the choice of experimental approach (Newgard 2017). Typically, untargeted metabolomics experiments generate substantial volumes of complex data requiring specialized computational processing and interpretation methods. Data interpretation software should ideally be capable of background noise elimination, peak identification and alignment, and peak normalization. While commercial and public domain software packages attempt to perform some of these tasks, there is no universal software for data extraction and analysis software. In metabolomics, hundreds of metabolites are detected and routinely analyzed. The complexity and magnitude of data produced from metabolomic studies necessitate the use of computational methods to analyze the data and elicit potential trends.

Artificial Intelligence (AI), both as a concept and research field, has gained attention across the twenty-first century. With its various applications in understanding the structures or trends in vast amounts of data collected or generated from modern high-throughput experiments, AI and machine learning (ML) offer countless possibilities. ML is used to develop models that can tackle large-scale data and, through learning, can solve complex problems. ML algorithms are fundamentally based on the ability to build mathematical models from a group of sample data (Dhall, Kaur, and Juneja 2020). Typically, a dataset used for developing a machine learning model is divided into a training subset, for example, comprised ∼70% of the available data and used in the ML algorithm to build a model and make predictions, and a testing subset, for example, ∼30% of the data used to provide an unbiased evaluation of the final model from the training step. Often, an intermediate validation step is added to assist in determining the most accurate model and obtaining optimal model hyper-parameters. In this instance, the data can be divided through a 60-20-20 split, where 20% of the data can act as an additional validation set. The initial learning process requires extensive data to allow the ML algorithm more opportunities to learn and improve the model. The ability of the algorithm to learn is formally through a mathematical function that maps specific inputs to certain outputs. The training dataset is used to guide the algorithms to make predictions without being explicitly programmed. This is achieved through a series of operations, where learning is made on the basis of weights and biases that will eventually make predictions in a finite number of steps (Cohen 2021). Having experienced the training dataset where the algorithm was able to learn and build a general model, the next step is testing the model’s performance on an independent dataset that contains previously unseen data and producing sufficiently accurate predictions. Predictions are based on the algorithm’s ability to assign each input to the chosen statistical representation defined by the user. The better the algorithm can learn from the input data provided, the more accurate the algorithm can produce predictions (Antonakoudis et al., 2020; Deepthi et al., 2020).

Constructing an ML model requires a series of steps: 1) Defining the training dataset: it involves identifying the type of data to be used as the training dataset; the input data would change depending on the problem that needs to be addressed. 2) Gathering the training dataset: a representation of the real-world use requires a set of inputs that will address the problem under investigation. 3) Input feature representation: The learned function’s accuracy strongly depends on how the input object is represented. Input objects are transformed into feature vectors, which have several descriptive features. The number of features must be sufficient to contain enough information to predict the output accurately and not too large to affect the dimensionality. 4) Determining the type of algorithm to be used: this is the algorithm that will be used to fit the data during the testing/training phase into a model. The choice of the algorithm depends on several factors, including the question the analysis is trying to answer, the data, and the ML category used, i.e., supervised learning, unsupervised learning, reinforcement learning, and semi-supervised learning (it is expanded upon later). 5) Training the algorithm: running the algorithm on the gathered training dataset; this step might require additional user input depending on the choice of the algorithm. Cross-validation can be used to adjust hyper-parameters (variables that determine how the algorithm is trained, e.g., learning rate, number of branches, clusters, and epochs) and optimize performance on a subset of the training set. 6) Validation: the training phase is often followed by a validation step to fine-tune the hyper-parameters of the classifiers. This validation step is independent of the cross-validation performed on the training set and uses a separate validation dataset. Validation is typically necessary to compare the performance of the different candidate classifiers: it is used to obtain performance parameters, including accuracy, sensitivity, and specificity of the models, and to estimate the models’ prediction error or bias. The model with the best performance on the validation set is then chosen to move forward to the testing phase. 7) Testing and evaluation: after hyper-parameter adjustments and learning, the accuracy of the learned function is assessed through the performance of the algorithm on an entirely new testing dataset, independent of the training and validation dataset (Figure 1: Principles of Metabolomics experimental design and associated ML workflow.).

Model performance assessment is an important step in properly evaluating the validity of a model’s predictions and deciding which model is best for a given problem. Model assessment methods are varied, depend on the characteristics of the problem, and can include a process known as hyper-parameter tuning, where they can be used to control the learning process of the model. The most commonly used assessment methods for classification problems are accuracy (Gajda and Chlebus 2022), cross-entropy (Boubezoul, Paris, and Ouladsine 2008; Gajda and Chlebus 2022), area under the curve (AUC) (Airola et al., 2011; Yala et al., 2019; Gajda and Chlebus 2022), while for regression analysis, mean squared error (Bellet, Habrard, and Sebban 2013), mean absolute error (Airola et al., 2011; Bellet, Habrard, and Sebban 2013) and root mean squared error (Nguyen et al., 2019) are more commonly employed. However, other performance metrics are available, including variance and $R^{2}$ coefficient, to name a few. Determining model specificity (the ability of a model to identify true negatives correctly) and sensitivity (the ability of a model to correctly identify true positives) (Trevethan 2017) are additional methods that can inform researchers and apply some context to the data under investigation (Parikh et al., 2008; Trevethan 2017).

With every newly discovered metabolite, the field of metabolomics has grown, allowing for a comprehensive assessment of biological specimens and their associated compounds. This improved understanding of the human body at the molecular and biochemical levels is crucial in aiding disease diagnosis and therapeutic development (Gowda et al., 2008). Over the years, the significant contribution of AI and associated applications in various biomedical fields has grown, demonstrating the application of ML in disease prediction and diagnosis of multiple diseases, including cardiovascular disorders, cancer, and rare genetic diseases.

**FIGURE 2:**
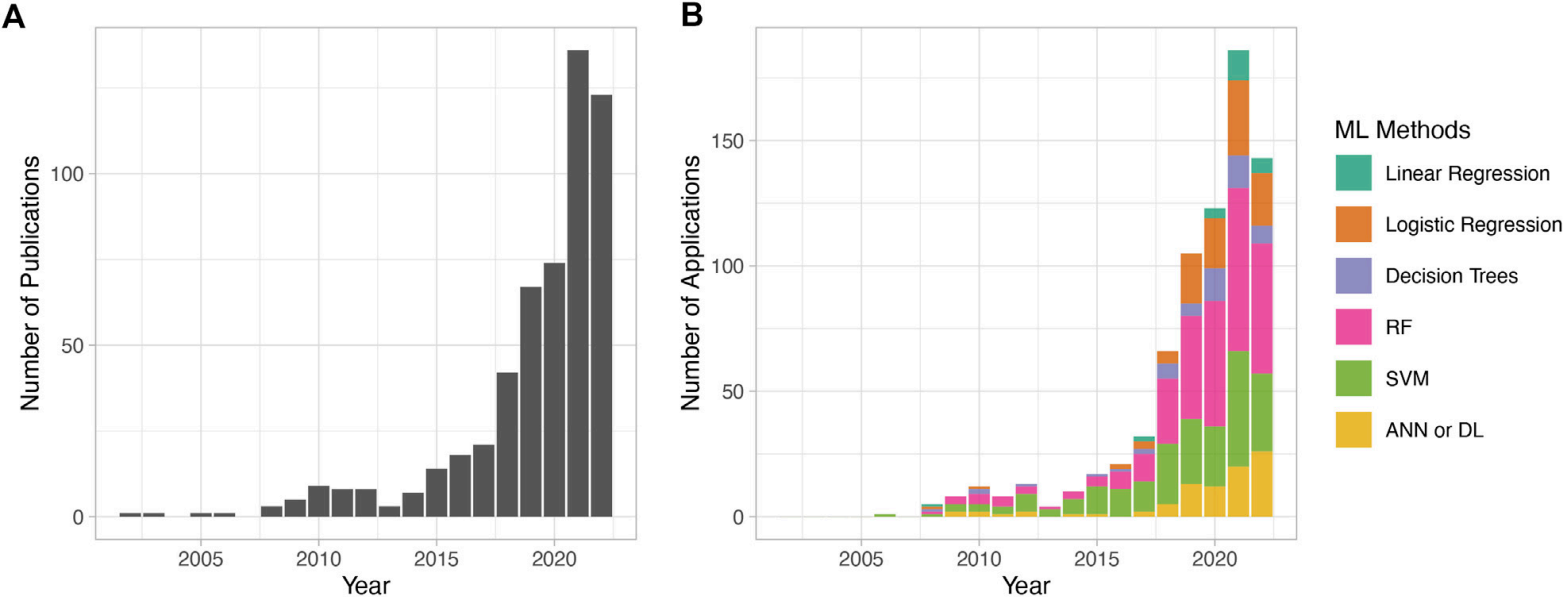
Metabolomic publications using machine learning in data analytics over the past 2 decades. PubMed was searched using the keywords “metabolomics” and “machine learning” from 2002 to 2022. Results were manually filtered to remove review articles and irrelevant publications. The counted publications include studies that use any of the mentioned ML algorithms in the context of metabolomic analysis, including classification problems, biomarker discovery, peak identification, metabolomic data analysis tools, and others. Only ML algorithms employed for disease model building are considered. **(A)** The total number of publications per year. **(B)** The number of publications using ML methods per year. The *y*-axis in **(A)** and **(B)** are different because in **(B)**, it indicates only the ML methods discussed in this review. The total number of publications across panels **(A)** and **(B)** varies because publications often utilize multiple ML algorithms.

In 2019, an editorial published in *Nature* titled “Why the metabolism field risks missing out on the AI revolution” expressed concern with the lack of momentum in AI-assisted applications in the field of metabolic research as opposed to other areas, such as genetics, for example. The curation of high-quality datasets, as well as the collective efforts of various institutions and funding bodies over the past few years, has increased the number of AI-assisted metabolomics studies. The number of metabolomic publications with AI and ML-based methods has been consistently on the rise, with very few publications (∼1/year) in the early 2000s, steadily rising to reach ∼150 publications in 2021, and the number of ML-assisted publications in 2022 promising to surpass this. The most used ML methods in metabolomic studies in the past years are RF, SVM, logistic regression, and, more recently, DL (Figure 2).

The integration of metabolomics with analytical ML techniques can be used to answer questions that other omics approaches cannot answer alone (Gowda et al., 2008; Graham et al., 2018; Turi et al., 2018; Jendoubi 2021). Here, we discuss major ML approaches for analyzing metabolic profiles, focusing on biomarker discovery and disease diagnosis.

## Types of machine learning algorithms

**FIGURE 3:**
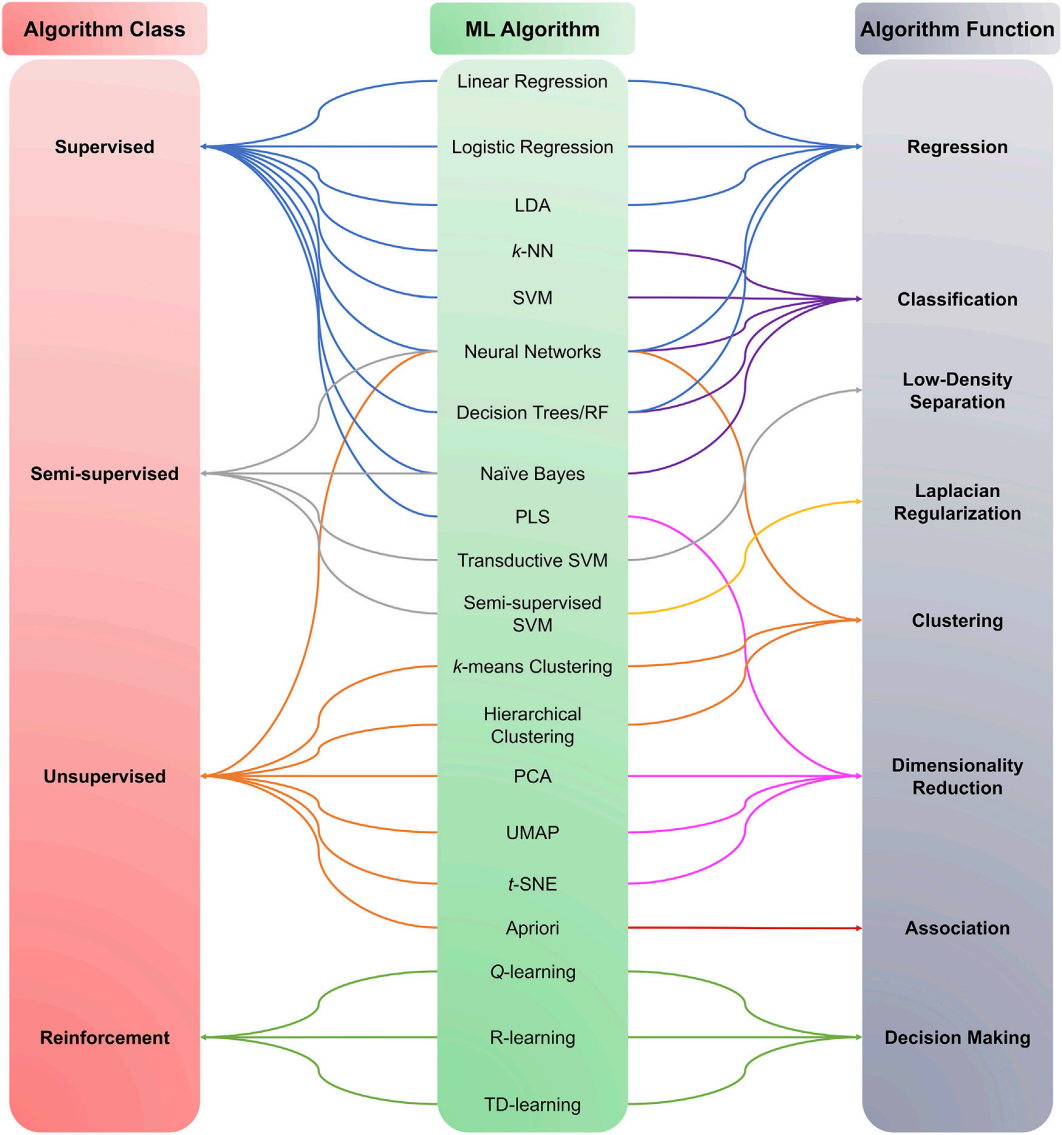
Machine learning algorithms categories. ML algorithms are divided into four main classes: Supervised, Unsupervised, Semi-supervised, and Reinforcement learning. The category choice depends on the type and nature of the data under investigation, i.e., labeled or unlabelled data.

ML algorithms can be used to analyze ever-increasing amounts of generated and accumulated data. ML algorithms are traditionally divided into supervised, unsupervised, semi-supervised, and reinforcement learning (Figure 3). For the purposes of this review, we focus on ML algorithms used in metabolomic studies, mainly supervised and unsupervised algorithms. The algorithms highlighted in the following sections do not exclusively belong to any of the mentioned ML categories; rather, the same algorithms can be used for multiple learning categories (e.g., *k*-Nearest Neighbor can be used in supervised and unsupervised learning).

## Machine learning categories

### Supervised learning

Supervised learning involves inferring a function from a labeled dataset input and a specific expected result (output), i.e., an input-output pair. With data containing continuous values, linear regression analysis is commonly used for objectives such as forecasting, prediction, and process optimization (Biswas, Saran, and Wilson 2021). Logistic regression is used with the classification into two categories. Classification for more than two categories can be performed using Support Vector Machines (SVM), decision trees, Random Forest (RF), and other methods (refer to Figure 3).

### Unsupervised learning

In unsupervised machine learning, the algorithm learns patterns from unlabeled data. The algorithm takes a dataset with only inputs and attempts to find a structure in the data by grouping or clustering the data points (Dhall, Kaur, and Juneja 2020). Unlike supervised learning, where the algorithm learns from data that has been labeled, classified, or categorized, unsupervised algorithms identify trends or commonalities in the data and respond based on the presence or absence of these commonalities in the data. This analysis can have various goals, from identifying hidden data trends to reducing redundancy, i.e., dimensionality reduction using Uniform Manifold Approximation and Projection (UMAP) (McInnes, Healy, and Melville 2018) and t-distributed stochastic neighbor embedding (t-SNE) (Van Maaten and Hinton 2008), or grouping together similar data (Dhall, Kaur, and Juneja 2020), i.e., clustering. Examples of unsupervised algorithms include *k*-means clustering, hierarchical clustering, anomaly detection, neural networks (NN), principal component analysis (PCA), independent component analysis (ICA), and *apriori* algorithms.

### Semi-supervised learning

Semi-supervised learning falls between unsupervised and supervised learning. It combines a small amount of labeled data with a large amount of unlabeled data during the training process and uses context to identify data patterns (Dhall, Kaur, and Juneja 2020). For example, this method can be used for classification problems that require a supervised learning algorithm to achieve the end goal; however, it would not require extensive labeling. It is faster than supervised learning because it involves a mixture of labeled and unlabeled data. Examples include generative models, low-density separation, Laplacian regularization, and heuristic approaches. This approach is not commonly used in the field of metabolomics, with few published applications (Libbrecht and Stafford Noble 2015; Migdadi et al., 2021; Abram and Douglas, 2022; Iqbal et al., 2022).

### Reinforcement learning

This method was adopted to direct unsupervised ML by rewarding desired behavior and punishing undesired ones. Positive feedback strengthens the model’s ability to connect target inputs and outputs (Dhall, Kaur, and Juneja 2020). Examples include Monte Carlo, Q-learning, State–action–reward–state–action (SARSA), Q-learning Lambda, SARSA-Lambda, and Deep Q-Learning (DQN), to name a few. Reinforcement learning is often converged around fields such as game theory, operations research, and swarm intelligence, as they are highly dependent on using robotics.

**FIGURE 4:**
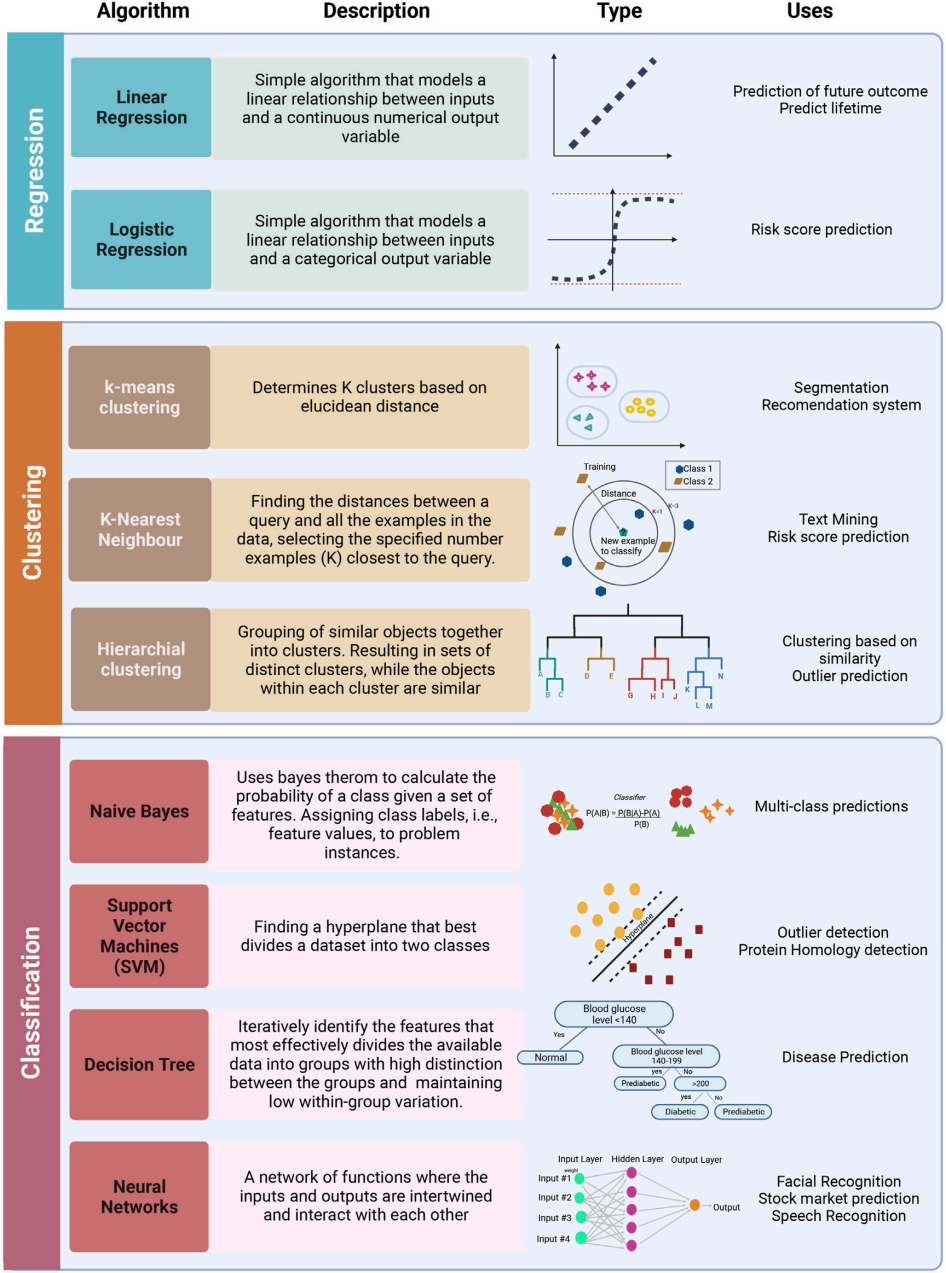
Representation of most commonly used ML algorithms with functional categorization accompanied by graphical representations of each algorithm and some potential applications. The most frequently used algorithms can be grouped into regression (linear and logistic), clustering (k-means, k-NN, hierarchical clustering, NN), and classification (Naive Bayes, SVM, Decision trees). Created with BioRender.com.

On the functional level, different ML algorithms are mainly geared toward solving regression, clustering, or classification problems. A representation of different ML algorithms with functional categorization is depicted in Figure 4, and brief descriptions of the most commonly used ML algorithms are indicated in Table 1. Supervised ML algorithms are by far the most commonly used in the field of metabolomics. For this review, six algorithms centering around supervised learning are highlighted in the following section, and the application of these algorithms to metabolomic data will be expanded upon.

**TABLE 1:** Key applied machine learning algorithms.

Algorithm	Description
Linear regression	A linear approach to model a relationship between dependent and independent variables (Schneider, Hommel, and Blettner 2010)
Logistic Regression	Models the probability of an event ocuring out of two alternatives by defining the logarithmic odds of the event is a linear combination of independent variables (Stoltzfus 2011)
*k*-means clustering	Partitions data into groups of similar kinds of items by finding the similarity between the items using euclidean distance. (MacQueen 1967)
Partial Least Squares (PLS)	Reduces the dimensionality of correlated variables to a smaller set of variables that can then be used as predictors. Used when there is a high number of colinear variables. (Garthwaite 1994)
Linear Discriminant Analysis (LDA)	Finds a linear combination of features that can separate two or more object classes. Uses a generalization of Fischer’s linear discriminant. (Riffenburgh 1957)
Boosting algorithms	Involves training a sequence of weak models, where each model compensates for the weakness of its predecessors. Thereby improving the overall predictive ability of the model. (Kearns and Valiant 1989)
Support Vector Machines (SVMs)	Based on finding a hyperplane that best divides a dataset into two classes (Boser, Guyon, and Vapnik 1992; Ben-Hur et al., 2002)
Naïve Bayes	Assigns class labels, i.e., feature values to problem instances (Bzdok, Altman, and Krzywinski 2018; Hastie et al., 2009).
*k*-Nearest Neighbors (*k*-NN)	Finding the distances between a query and all similar examples in the dataset, selecting the specified number of examples (K) closest to the query, when used for classification, the most frequent labels are counted and when used for regression, the labels are averaged (Altman 1992)
Decision Trees	Uses a tree-like model of decisions and consequences to predict the value of a target variable by learning simple decision rules from available data features (Shalev-Shwartz and Ben-David 2014)
Random Forest (RF)	Builds on the concept of multiple decision trees and takes the majority for classification and the average for regression (Hastie et al., 2009; Ho 1995)
Principal Component Analysis (PCA)	A dimensionality reduction technique that projects data onto a subspace of lower dimension that is able to retain the most variance among the data points. (Wold, Esbensen, and Geladi 1987; Jolliffe 2005)
Neural Networks (NN)	A network of functions where the inputs and outputs are intertwined and interact with each other (Hinton and Salakhutdinov 2006)

## Machine learning algorithms

### Regression analysis

Regression analysis is a group of statistical procedures used to estimate the relationship between a dependent variable (outcome or response) and one or more independent variables (predictors, covariates, or features). This method of statistical analysis progressed from the least-squares method to the regression. It can be used in a variety of fields. In order to interpret the output in real-world relationships, a number of assumptions are made, such as that the sample is representative of the entire population and that no errors occurred when measuring the independent variables (Vetter and Schober 2018). Regression analysis is used for two distinct purposes: inferring causal relations between the variables under investigation and prediction (Baumgartner, Böhm, and Baumgartner 2005).

### Linear regression

Linear regression models the relationship between dependent and independent variables by fitting a straight line (linear equation) to the observed data (Schneider, Hommel, and Blettner 2010). Predictions based on linear regression are simple: data trend is observed, then a prediction is made on the basis of that trend (Casson and Farmer 2014; Vetter and Schober 2018). While not all data follow a linear trend, linear regression is often the first attempt used to understand data and for predictive analyses.

### Logistic regression

A statistical model used to predict a binary outcome (one scenario out of two possible alternatives) based on a set of independent variables (those that influence the outcome) using a logarithmic odds scale (Stoltzfus 2011). Typically, logistic regression analysis is used for classification purposes and when dealing with binary outcomes i.e., two categories.

### Decision trees

A statistical decision support tool that uses a tree-like model of decisions and possible consequences. Each tree is similar in structure to that of a flowchart. Each node represents a test, e.g., taking a vitamin, each subsequent branch represents the outcome of the test, i.e., “yes” or “no” for taking the vitamin, and each leaf node represents a class label (Shalev-Shwartz and Ben-David 2014; Kamiński, Jakubczyk, and Szufel 2018). Decision trees consist of three types of nodes: decision, chance, and end nodes (Kamiński, Jakubczyk, and Szufel 2018). Decision trees are constructed to iteratively identify the feature that most effectively divides the available data into groups with a high distinction between the groups in terms of outcome while maintaining a low within-group variation.

### Random forest (RF)

A statistical classification method composed of an *assembly* of many decision trees constructed during the training phase. Generally outperforming decision trees as they correct the observed overfitting. New objects are classified based on the attributes of the data. Each tree is classified and gives a vote for the chosen attribute. When used for classification, the classification with the most votes is chosen, and when used for regression purposes, the average votes are used (Hastie et al., 2009; Dhall, Kaur, and Juneja 2020). RF models are among the most frequently used algorithms for prediction or classification purposes, with various omics applications from understanding the human gut microbiome, differentiating between healthy and disease metabolome, investigating the pregnancy metabolome, cancer diagnosis to the more recent COVID-19 diagnosis and classification of COVID-19 severity. Key studies using these ML algorithms for metabolomic understanding will be highlighted later.

### Support vector machines (SVM)

**FIGURE 5:**
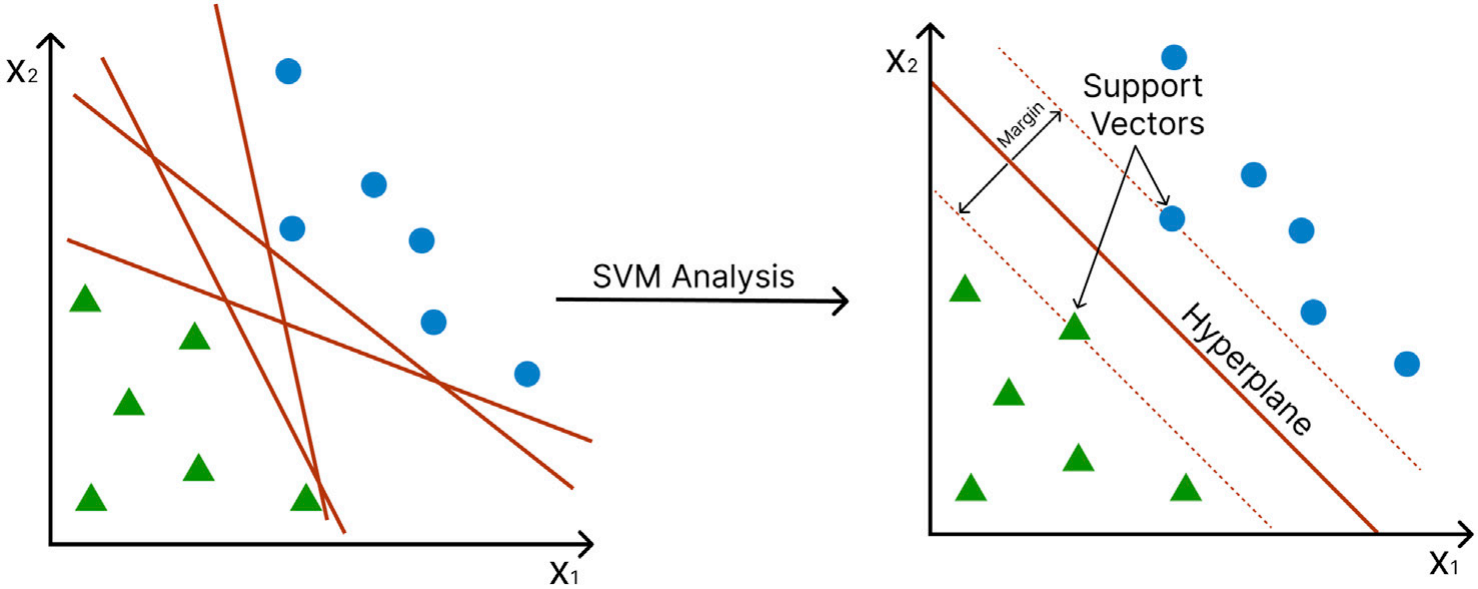
Support Vector Machines (SVM) construct a hyperplane to separate data into two classes. Axes represent different features. Green triangles and blue circles represent different conditions (e.g., disease vs. control). The margin (red dotted line) is the distance between the hyperplane and the support vectors (the nearest data point of each class).

Proposed in 1992 by Boser, Guyon, and Vapnik, SVMs (Boser, Guyon, and Vapnik 1992) has been popular classification tools in many fields, including bioinformatics and biological data analysis in general (Saeys, Inza, and Larrañaga 2007). SVMs split training observations into two classes by constructing a hyperplane, a decision boundary that separates the data points into two classes. The distance between the hyperplane and the nearest data points of each class is called the margin, and the points onto which this margin hits are called the “support vectors”. The SVM is constructed so that the margin on either side of the hyperplane is maximized (Figure 5) (Vapnik 2006). In many cases, the data points cannot be fully segregated. Here, the SVM will try finding a “soft margin” that allows the misclassification of a few points while minimizing the cost of the training points that are on the wrong side of the classification boundary (Cortes and Vapnik 1995).

**FIGURE 6:**
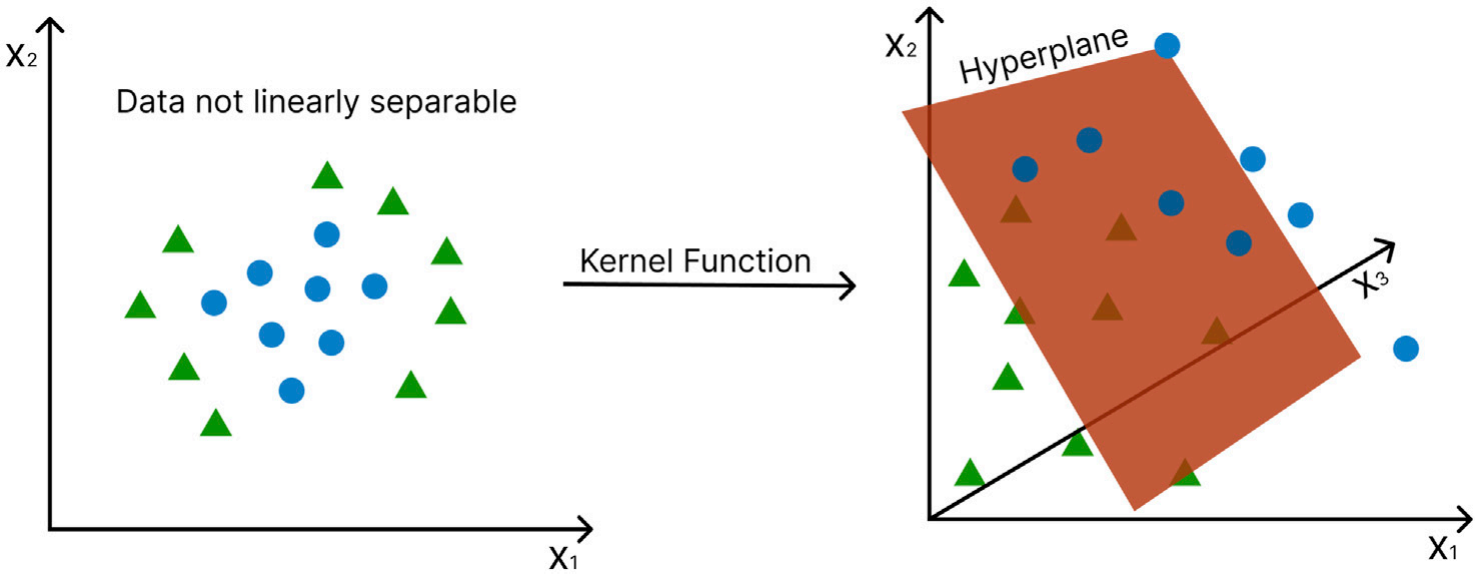
The “kernel trick” - non-linearly separable data points are mapped into a higher dimensional feature space in which they become linearly separable. Axes represent different features. Green triangles and blue circles represent different conditions (e.g., disease vs. control). The hyperplane, in this case, becomes a two-dimensional plane.

In the case of data that are not linearly separable, the data points are mapped into a higher dimensional feature space in which they become linearly separable (Cortes and Vapnik 1995) (Figure 6). This is known as the “kernel trick” and gives SVMs major advantage over other statistical multivariate methods, such as PCA, Partial Least Squares (PLS), and Orthogonal Projections to Latent Structures (OPLS), which cannot be applied to nonlinear cases. A variety of different kernel functions can be employed to transform the data, including the linear kernel, polynomial kernel, sigmoid kernel, and Gaussian radial basis function (RBF) kernel (Powell 1987), (Broomhead and Lowe 1988). A major drawback of SVM is that it is natively restricted to binary classification problems, i.e., it can only discriminate between two classes. However, it does not scale well with large datasets because of its computational complexity.

It is often beneficial to perform feature selection before training multivariate algorithms, such as SVMs, by only selecting subsets of features, in the case of metabolites, on which supervised learning is employed (Guyon 2003). Reducing the number of variables used for model building can simplify the interpretability of the data analysis results and prevent overfitting, which is often caused by non-informative input features (Liu and Motoda 2012). Feature selection methods have been reviewed elsewhere (Miao and Niu 2016). Popular feature selection methods used with SVM models in metabolomics studies include recursive feature elimination (RFE) (Guan et al., 2009; R. Shen et al., 2018), L1 norm SVM (Zhou et al., 2010) (Guan et al., 2009) (Zhou et al., 2010) and variable importance in projection (VIP) ((Zhang et al., 2018), (Cheng et al., 2019) (Z. Chen et al., 2021)).

### Deep learning (DL)

Deep learning (LeCun, Bengio, and Hinton 2015) has risen to prominence as the most popular type of machine learning algorithm recently. It uses artificial neural networks (ANN) to construct complex relationships relating input variables to the outcome, advancing classifier performance beyond typical machine learning techniques, particularly in circumstances involving large-scale datasets with high dimensionality. The potential of deep learning is endless; however, it is an intensive process that requires considerable computational power, and its results are often difficult to interpret. In the case of metabolomics studies, it is difficult to evaluate from the model, which features affect classification the most. Deep learning’s recent success has been fueled by an increase in computing power—particularly the introduction of graphics processing units, or GPUs —, as well as the availability of large-scale data sets to use for training the models.

Although there are applications of unsupervised deep learning, including autoencoders (Rumelhart, Hinton, and Williams 1985; Hinton and Salakhutdinov, 2006; Hinton and Salakhutdinov 2006) and generative adversarial networks (Goodfellow et al., 2014 (Goodfellow et al., 2014)), in this review, we focus on supervised deep learning.

**FIGURE 7:**
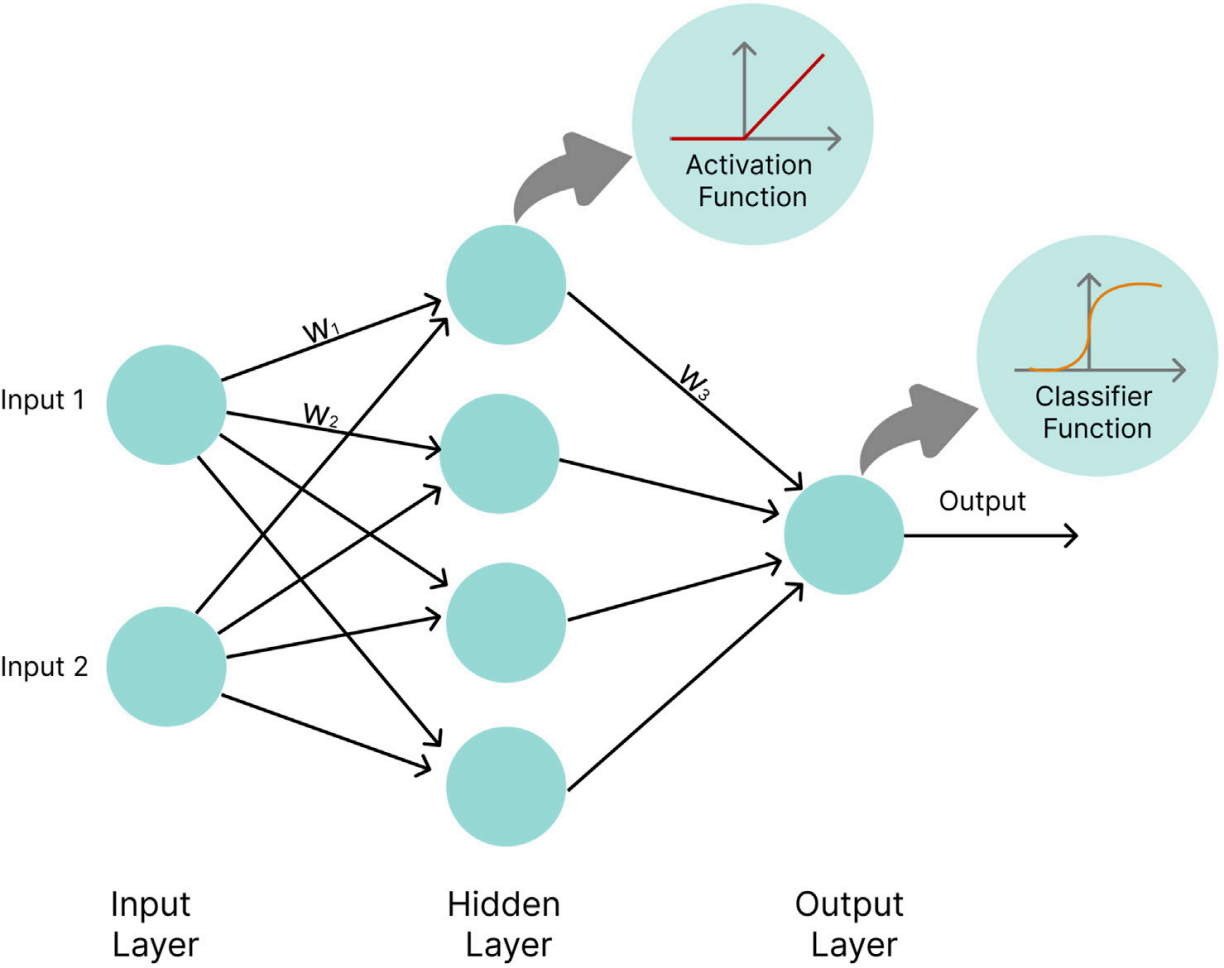
Basic neural network architecture. Circles represent neurons. w_1_, w_2,_ and w_3_ represent weights by which values calculated inside neurons are multiplied before being passed on to the next layer. In the hidden layer neurons, values are passed into an activation function (e.g., the ReLU function), while the output layer neuron applies a classifier function (e.g., the Softmax function) to input values.

An artificial NN is composed of units, termed neurons, that combine multiple inputs and produce a single output. The network approximates the functions that link inputs (e.g., gene expression levels, metabolite concentrations) to desired outputs (e.g., disease risk). Neurons are organized into several layers, with an input layer, an output layer, and intermediate layers, called “hidden layers” (LeCun, Bengio, and Hinton 2015). The variables from the input layer are multiplied by specific values called weights and fed into the neurons of the first hidden layer. Each neuron takes the input, and applies a nonlinear activation function to it, such as sigmoid (Narayan 1997) or rectified linear unit (ReLU) (Glorot et al., 2011), and modifies the outcome by adding a bias to it. The output is then passed on to the next hidden layer. Finally, the outputs of the hidden layers are linearly combined in the output layer and often passed through a classifier function, e.g., a Softmax function, to produce an output value. During supervised NN training, the tunable parameters of the network, i.e., the weights and biases, are optimized so that the distance between the network’s computed outcome and the experimentally determined outcome is minimized (Figure 7).

**FIGURE 8:**
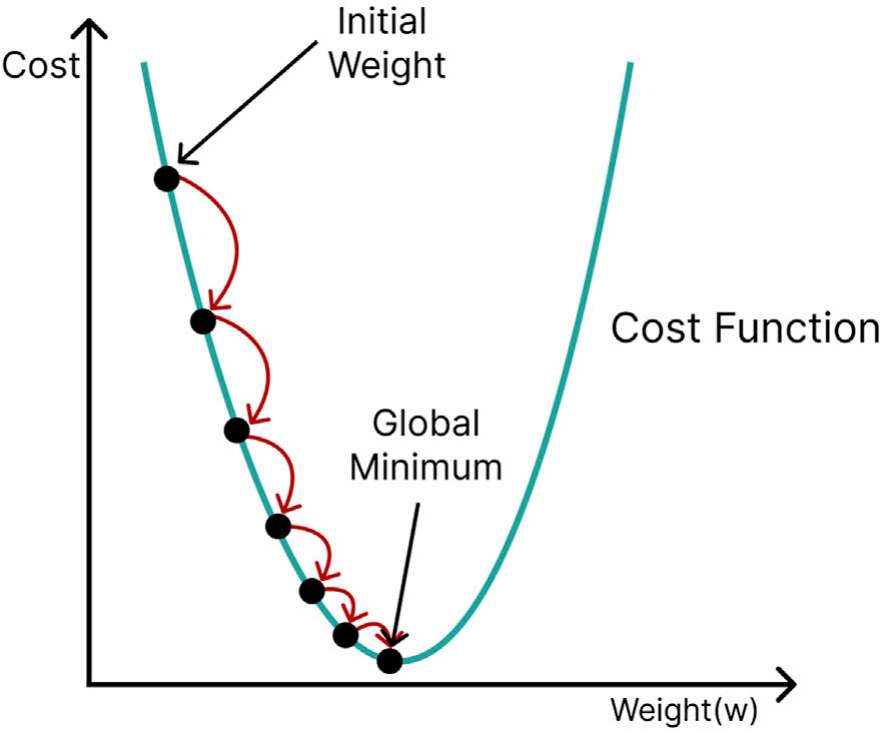
Gradient descent; initial network parameters (weights and biases) are adjusted in a direction that travels down the slope of the cost function (green curve) until the minimum is reached.

Weights and biases are usually randomly initialized in an artificial neural network and then gradually optimized with the aid of a backpropagation algorithm. A cost function (e.g., the sum of squared errors, cross-entropy) computes the difference between the network’s output and the desired output. The derivative of the cost function with respect to the weight can be used to evaluate how a slight change in a particular weight affects performance. The parameters of the network are adjusted in a direction that minimizes the cost. This process is termed gradient descent because it travels down the slope of the cost function in steps until, optimally, it reaches its global minimum (Figure 8). However, cost functions are often complicated in reality, with many local minima and saddle points to which gradient descent could converge. Since the slope in these regions is also zero, it is almost impossible to escape them. Stochastic gradient descent (Bottou 2010) offers a more efficient approach, in which only a subset (minibatch) of the training data is selected at random and used for cost minimization. Using different mini-batches for each calculation provides enough stochasticity to avoid getting stuck in local minima and saddle points, in addition to drastically reducing computation time and cost.

An artificial NN is considered ‘deep’ when it contains more than one hidden layer. It has been shown that a single hidden layer can approximate any function that maps input patterns to output patterns, given that sufficient neurons are employed (Cybenko 1989), (Hornik, Stinchcombe, and White 1989). However, using more hidden layers improves generalization and leads to more accurate modeling (LeCun, Bengio, and Hinton 2015). Some commonly used types of artificial NN include feed-forward NN, recurrent neural networks, convolutional neural networks (CNN), and deep Boltzmann machines. For an excellent review of NN types, refer to Min et al., 2016 (Min, Lee, and Yoon 2016); for potential applications, refer to (Mendez, Broadhurst, and Reinke 2019; Pomyen et al., 2020).

DL has only recently been used in the analysis of omics data, and the application of DL in metabolomics, in specific, is still emerging and comparatively low compared to other omics. Metabolomic studies that use DL algorithms are, therefore, much fewer than those that utuseilize other ML algorithms.

### Use of ML approaches in metabolomic studies

Recently, ML techniques have been used for the analysis of metabolomics data from numerous diseases. For the purposes of this review, we are focusing on key studies that used the aforementioned ML approaches in metabolomic investigations, categorized according to the conditions being studied. For examples of metabolomic studies using ML approaches, refer to Table 2 and Supplementary Table S1.

**TABLE 2:** Examples of metabolomics studies utilizing ML algorithms.

#	Author	Journal	Publication year	Area of investigation	ML algorithm used	Brief description	Findings	Doi
1	Shen et al	Cell	2020	COVID-19	Random Forest	Identification of severe COVID-19 cases based on molecular signatures of proteins and metabolites	Severity identification was conducted on 18 non-severe and 13 severe patients. Identified 29 important variables (22 proteins, 7 metabolites) - > Incorrect classification of 1 patient	doi: 10.1016/j.cell. 2020.05.032. Epub 2020 May 28. PMID: 32492406; PMCID: PMC7254001
							Model was tested on an independent cohort of 10 patients - > all severe patients correctly identified except 1	
2	Han et al	Nature	2021	Human gut microbiota	Random Forest	Identification of distinct metabolites to differentiate between different taxonomic groups	The model revealed subsets of chemical features that are highly conserved and predictive of taxonomic identification	doi: 10.1038/s41586-021-03707-9. Epub 2021 Jul 14. PMID: 34262212; PMCID: PMC8939302
							e.g., over-representation of amino acid metabolism	
3	Liang et al	Cell	2020	Human pregnancy metabolome	Linear regression	Untargeted metabolomic profiling and identification of metabolic changes in human pregnancy	Detection of many of the previously reported pregnancy-associated metabolite profiles	doi: 10.1016/j.cell. 2020.05.002. PMID: 32589958; PMCID: PMC7327522
							>95% of the pregnancy associated metabolites are previously unreported	
4	Hogan et al	EBioMedicine	2021	Influenza	Gradient boosted decision trees and random forest	Untargeted metabolomics approach for diagnosis of influenza infection	Untargeted metabolomics identified 3,318 ion features for further investigation	doi: 10.1016/j.ebiom. 2021.103546. Epub 2021 Aug 19. PMID: 34419924; PMCID: PMC8385175
							Described LC/Q-TOF method in conjunction with machine learning model was able to differentiate between influenza samples (pos/neg) with sensitivity and specificity over 0.9	
5	Bifarin et al	J Proteome Res	2021	Renal Cell Carcinoma	Partial Least Squares	A 10-metabolite panel predicted Renal Cell Carcinoma within the test cohort with 88% accuracy	A total of 7,147 metabolites were narrowed down to a series of 10 and tested with 4 ML algorithms all of which were able to correctly identify RCC status with high accuracy in the test cohort	doi: 10.1021/acs.jproteome.1c00213. Epub 2021 Jun 23. PMID: 34161092
					Random Forest Recursive feature elimination			
					K-NN			
6	Tiedt et al	Ann Neurology	2020	Ischemic Stroke	Random Forest classification	Identified 4 metabolites showing high accuracy in differentiating between Ischemic stroke and Stroke Mimics	Levels of 41 metabolites showed significant association with Ischemic stroke compared to controls. Top 4 metabolites show high accuracy in differentiating between stroke and mimics	https://doi.org/10.1002/ana.25859
					Linear discriminant analysis			
					logistic regression			
					K-NN			
					naive Bayes			
					SVM			
7	Liu et al	Mol Metabolite	2021	Diabetic kidney disease	Linear discriminant analysis	Serum integrative omics provide stable and accurate biomarkers for early warning and diagnosis of Diabetic Kidney Disease	combination of a2-macroglobulin, cathepsin D, and CD324 could serve as a surrogate protein biomarker using 4 different ML methods	doi: 10.1016/j.molmet. 2021.101,367. Epub 2021 Nov 1. PMID: 34737094; PMCID: PMC8609166
					SVM			
					Random Forest			
					Logistic regression			
8	Oh et al	Cell Metab	2020	Cirrhosis	Random Forest	Comparison of the dysregulation between gut microbiome in differentiating between advanced fibrosis and cirrhosis	Identified a core set of gut microbiome that could be used as universal non-invasive test for cirrhosis	doi: 10.1016/j.cmet. 2020.06.005. PMID: 32610095; PMCID: PMC7822714
9	Delafiori et al	Anal Chem	2021	COVID-19	ADA tree boosting	Combine ML with mass spectrometry to differentiate between COVID-19 in plasma samples within minutes	Diagnosis can be derived from raw data with diagnosis specificity 96%, sensitivity 83%	doi: 10.1021/acs.analchem.0c04497. Epub 2021 Jan 20. PMID: 33471512; PMCID: PMC8023531
					Gradient tree boosting			
					Random forest			
					partial least squares			
					SVM			
10	Jung et al	Biomed Pharmacother	2021	Coronary artery disease	Logistic regression	10-year risk prediction model based on 5 selected serum metabolites	provided initial evidence that blood xanthine and uric acid levels play different roles in the development of machine learning models for primary/secondary prevention or diagnosis of CAD. Purine-related metabolites in blood are applicable to machine learning model development for CAD risk prediction and diagnosis	doi: 10.1016/j.biopha. 2021.111,621. Epub 2021 May 10. PMID: 34243599
11	Wallace et al	J Pathol	2020	Cancer	Linear discriminant analysis	Comparison between metabolic profile of tumor patients and the predictive ability of machine learning algorithm to interpret metabolite data	Application of machine learning algorithms to metabolite profiles improved predictive ability for hard-to-interpret cases of head and neck paragangliomas (99.2%)	doi: 10.1002/path.5472. Epub 2020 Jul 1. PMID: 32462735; PMCID: PMC7548960
12	Kouznetsova et al	Metabolomics	2019	Bladder cancer	Logistic regression	Elucidate the biomarkers including metabolites and corresponding genes for different stages of Bladder cancer, show their distinguishing and common features, and create a machine-learning model for classification of stages of Bladder cancer	The best performing model was able to predict metabolite class with an accuracy of 82.54%. The same model was applied to three separate sets of metabolites obtained from public sources, one set of the late-stage metabolites and two sets of the early-stage metabolites. The model was better at predicting early-stage metabolites with accuracies of 72% (18/25) and 95% (19/20) on the early sets, and an accuracy of 65.45% (36/55) on the late-stage metabolite set.	doi: 10.1007/s11306-019-1,555-9. PMID: 31222577
13	Murata et al	Breast Cancer Res Treat	2019	Breast Cancer	Multiple logistic regression	Combinations of salivary metabolomics and machine learning methods show potential for non-invasive screening of breast cancer	Polyamines were identified to be significantly elevated in saliva of breast cancer patients	doi: 10.1007/s10549-019-05330-9. Epub 2019 Jul 8. PMID: 31286302
14	Liu et al	BMC Genomics	2016	Major Depressive Disorder	SVM	Identifying the metabolomics signature of major depressive disorder subtypes		
					Random Forest		∼80% accuracy in classification of melancholic depression	
							doi: 10.1186/s12864-016-2,953-2. PMID: 27549765; PMCID: PMC4994306	


## Cancer

### Ovarian cancer

In one of the earliest studies, Yu et al. developed an SVM classification model that achieved an average sensitivity of 97.38% and an average specificity of 93.30% for distinguishing cancer from healthy tissue, using a dataset provided by the National Cancer Institute containing serum metabolomic data from ovarian cancer and normal tissue (Yu et al., 2005).

The research group of Guan et al. also extensively studied ovarian cancer metabolites. In 2009, they constructed classifiers using linear and non-linear SVM to diagnose ovarian cancer from serum metabolites with over 90% accuracy, significantly better than a random classifier (Guan et al., 2009).

The same research team published in 2010 (Zhou et al., 2010) how they evaluated a customized fSVM algorithm (SVM for functional data classification (Rossi and Villa 2006)) coupled with ANOVA feature selection for detecting of ovarian cancer using serum metabolites. One of the tested models achieved 100% accuracy in split validation and 98.9% in leave-one-out cross-validation.

In a third study published in 2015 (Gaul et al., 2015), the authors were able to generate a further SVM model capable of identifying early-stage ovarian cancer with 100% accuracy, this time using a panel of sixteen serum metabolites selected by RFE. Eleven of the sixteen metabolites were identified, including phosphatidylinositol, as well as the lysophospholipids lysophosphatidylethanolamine and lysophosphatidylinositol.

Metabolomic analysis has also been found to predict ovarian cancer recurrence. An SVM prediction model was employed by Zhang et al. with ten significant plasma biomarkers, yielding area under the curve (AUC) values reaching 0.964 (Zhang et al., 2018). The results showed a clear clinical advantage over the most commonly used clinical biomarker, CA125, which by contrast, produced an AUC value of only 0.6126.

### Breast cancer

An interesting metabolomics study on breast cancer by Henneges et al. focused on modified nucleosides (degradation products of cellular RNA metabolism) and ribosylated metabolites in urine samples (Henneges et al., 2009). From a set of 35 pruned metabolites, 44 pairwise combinations of metabolite features were employed for SVM-based analysis. The sensitivity and specificity of this model were 83.5% and 90.6%, respectively. S-adenosylhomocysteine (SAH) was the most commonly recurring compound in the metabolite pairs, underlining its importance for RNA methylation in cancer pathogenesis.

In another study conducted on breast cancer samples, Alakwaa et al. demonstrated that DL could reliably predict estrogen receptor status (Alakwaa, Chaudhary, and Garmire 2018). The authors used feed-forward networks with a sigmoid activation function and a softmax classifier on a dataset containing 162 metabolites. The predictions were compared to traditional ML methods like RF, SVM, prediction analysis for microarrays (PAMs), generalized boosted models, recursive partitioning and regression trees (RPART), and linear discriminant analysis, with DL models displaying the highest accuracy (AUC 0.93). This DL method also identified eight unique metabolic pathways that seem to promote breast cancer. The study’s findings suggest that DL may be used to deduce the topology of affected biochemical pathways from a network analysis of a metabolomics data set.

The predictive abilities of five potential urinary biomarkers for breast cancer were evaluated by Kim et al. (Kim et al., 2010). Multivariate methods (linear and Gaussian SVM algorithms, decision trees, and RF) were shown to outperform univariate methods by about 6.6–12.7%. It is noteworthy, however, that the linear SVM model scored the lowest in specificity.

### Endometrial cancer

Cheng et al. (Cheng et al., 2019) applied 4 ML algorithms- SVM, Partial Least Square-Discriminant Analysis (PLS-DA), RF, and LR-to identify metabolomic biomarkers in cervicovaginal fluid for endometrial cancer detection. The SVM and RF techniques displayed the greatest accuracy of 78% (75% sensitivity and 80% specificity) in the testing dataset.

### Hepatocellular carcinoma

Xue et al. (Xue et al., 2008) applied stepwise discriminant analysis (SDA) and SVM algorithms to identify a set of 13 serum metabolites to distinguish between patients with hepatocellular carcinoma and healthy controls with 75% accuracy. The metabolites included carbohydrates, amino acids, fatty acids, cholesterol, and low molecular weight organic acids.

### Lung cancer

A more recent study used SVMs with untargeted lipidomics to identify features most important for early-stage lung cancer detection (Wang et al., 2022). Lung plasma lipidomic profiling was carried out on 311 participants using mass spectrometry. Using SVM feature selection, nine lipids were chosen for developing a liquid chromatography-mass spectrometry-based targeted assay. The authors validated the ability of these nine lipids to detect early-stage cancer across multiple independent cohorts, including a hospital-based lung cancer screening cohort of 1,036 participants and a prospective clinical cohort containing 109 participants, in which the assay reached more than 90% sensitivity and 92% specificity. The selected lipids were also shown to be differentially expressed in early-stage lung cancer tissues *in situ*. This assay, which the authors named “Lung Cancer Artificial Intelligence Detector,” shows promise for the early detection of lung cancer and large-scale screening of high-risk populations for cancer prevention.

### Squamous cell carcinoma

In their 2019 study, Hsu et al. uncovered potential metabolic biomarkers for oral cavity squamous cell carcinoma (Hsu et al., 2019). They constructed a three-marker panel consisting of putrescine, glycyl-leucine, and phenylalanine, using an SVM model that can discriminate cancerous from adjacent non-cancerous tissues with high sensitivity and specificity based on receiver operating characteristic (ROC) analysis.

RF and SVM also demonstrated favorable results in the identification of esophageal squamous cell carcinoma tissue based on differential metabolites (Z. Chen et al., 2021). Among the three models evaluated, RF had the highest predictive performance (100%), but required more computational time (8.99 s), compared to PLS and SVM models, which showed similar predictive performance (95%) and similar computational time (1.27 s and 1.11 s). It is of note, however, that the three models prioritized different features.

### Non-Hodgkin’s lymphoma

Bueno Duarte et al., 2020, identified a panel of 18 urine metabolites that can differentiate diffuse large B-cell lymphoma patients from healthy individuals with 99.8% accuracy using an SVM model (Duarte et al., 2020).

### Renal cell carcinoma

In another cancer study, Bifarin et al. (Bifarin et al., 2021), identified candidate urine metabolic panels for renal cell carcinoma (RCC) as a noninvasive diagnostic assay. Information from patients and controls was gathered and divided into the model and test cohorts. Multiple ML algorithms were used to test the predictive ability. These include RF, KNN, linear kernel SVMs, and RBF kernel SVMs. A total of 7,147 metabolomic features were identified from the NMR and MS platforms. These were then merged and filtered to only those that showed a greater than 1-fold change between the RCC and control samples, and highly positively correlated features were removed. This hybrid model resulted in a selection of 10 metabolites for a panel. RCC status was tested across the used ML models, and all of them were able to predict RCC status accurately.

### Osteosarcoma

An RF classifier demonstrated superiority over an SVM model, with an accuracy of 85% *versus* 81% for the classification of osteosarcoma and benign tumor patients using both X-ray image features and serum metabolomic data (R. Shen et al., 2018).

## Non-cancer conditions

### Coronavirus disease (COVID-19)

With the onset of the COVID-19 pandemic, research groups across the globe conducted numerous investigations trying to understand if there was any biological reasoning behind disease heterogeneity, in terms of disease severity, presentation, and even mortality rate. For example, Chen et al. (B. Shen et al., 2020) combined proteomic and metabolomic profiles of 31 COVID-19 patients (18 non-sever, 13 severe) to create an ML molecular classifier, which was eventually able to identify potential blood biomarkers for severe COVID-19. The devised RF model identified 29 variables of priority (22 proteins, seven metabolites); this model had a 0.957 AUC in the training set. Subsequent testing of the model against an independent cohort of 10 patients revealed accurate identification of severe COVID-19 patients for all but one of the cohort. The incorrectly identified patients had potential confounding factors, i.e., age, long period of administration of non-traditional medicine, and several comorbidities. The generated classifier was again tested against a model with 29 randomly selected molecules. The randomly generated model exhibited low accuracy when compared with the classifier.

### Type 2 diabetes (T2D)

Shomorony et al. (Shomorony et al., 2020) identified a set of cardiometabolic biomarkers beyond the standard clinical biomarkers that can be used to stratify individuals into disease types and stages. Data features from 1,385 diverse modalities (microbiome, genetics, metabolome, advanced imaging) were collected from 1,253 self-assessed healthy individuals. A linear regression ML algorithm was used to identify whether there were any associated covariates. This was then validated through correlation analysis to identify any significant associations between features. Network analysis was performed to determine whether the identified modalities had biomarker signatures that corresponded to underlying biological systems. Finally, using the identified features, cluster analysis was performed to stratify participants into subsets consistent with their respective health status. The findings were validated in an independent cohort of 1,083 females. The authors highlighted several novel biomarkers in diabetes signature and gut microbiome health, i.e., 1-stearoyl-2 dihomo-linoleoyl-GPC and cinnamoyl glycine, respectively.

### Nonalcoholic fatty liver disease (NAFLD)

universal gut-microbiome signatures can be used to predict various diseases. This is true for Oh et al. (Oh et al., 2020) who used stool microbiome from 163 nonalcoholic fatty liver (NAFLD) disease patients and applied an RF ML algorithm with a differential abundance analysis to identify microbial and metabolomic signatures to detect cirrhosis and the authors were able to test the generated model and its ability to differentiate between cirrhosis and fibrosis. The model was able to correctly distinguish between the various stages of fibrosis with high accuracy AUC 0.85. The incorporation of further information into the model, i.e., serum AST levels, showed marked improvement in model performance with AUC 0.94.

Perakakis et al. trained models for the non-invasive diagnosis of non-alcoholic steatohepatitis (NASH) and NAFLD (Perakakis et al., 2019) from serum samples. SVM models including 29 lipids or combining lipids with glycans and/or hormones were shown to classify the conditions with 90% accuracy, and a 10-lipid-model could detect liver fibrosis with 98% accuracy.

### Acute myocardial ischemia (AMI)

A multilayer perceptron (MLP) neural network-based model achieved superior results in detecting acute myocardial ischemia (AMI) from serum metabolites in a rat model compared to several other classification algorithms, including SVM, RF, Gradient tree boosting (GTB), and LR (Cao et al., 2022). The model achieved accuracy of 96.67% in the rat model and 88.23% in predicting AMI type II in human autopsy cases of sudden cardiac death.

### Chronic kidney disease (CKD)

In an attempt to classify chronic kidney disease patients from serum metabolites, Guo et al. (Guo et al., 2019) constructed two NN; a two-layered fully connected multi-layer NN with MLP with 128 neurons in the hidden layer, and a three-layered CNN with 16 and 32 neurons in the two hidden layers, respectively. The MLP achieved accuracy of 90.4%, while the CNN reached accuracy of 90.6%. Both NNs, as well as an SVM model, were outperformed by an RF classifier with 100% accuracy. A possible reason is the rigorous feature reduction steps performed prior to model application; DL methods specialized in the analysis of high-dimensional data and in this study, from thousands of measured metabolites, only five were retained for the final models.

### Celiac disease

In one of the earliest and highly cited studies, metabolic signatures of celiac disease, detected by NMR, were used to construct an SVM model able to differentiate celiac disease patients from healthy controls with 83.4% accuracy using serum metabolites and 69.3% using urine metabolites. After a 12-month gluten-free diet, the same model correctly classified all but one of the patients as healthy (Bertini et al., 2009).

### Multiple Sclerosis (MS)

Waddington et al. used ML models including SVM, RF, k-NN, decision tree, and least absolute shrinkage and selection operator (LASSO) logistic regression to predict the tendency of multiple sclerosis patients treated with beta interferons to develop anti-drug antibodies (Waddington et al., 2020). Among the five classification models tested for predicting future immunogenicity from serum metabolomics data, SVMs were one of the most successful at differentiating between cases with and without drug resistance.

### Major depressive disorder

Metabolomic signatures associated with certain conditions may still persist after disease remission, as shown in a study by Hung et al., 2021. Eight plasma metabolites were identified as significantly differentially-expressed in patients with major depressive disorder (MDD) with full remission compared with healthy controls. These were then used to construct an SVM model capable of differentiating patients with MDD with full remission from healthy controls with predictive accuracy of nearly 85% (Hung et al., 2021).

### Schizophrenia

Chen et al. uncovered metabolic biomarkers that can differentiate between schizophrenia patients with violence and those without violence (X. Chen et al., 2020). RF and SVM analyses unveiled ten and five plasma metabolites, respectively. The common metabolites formed a biomarker panel, including the ratio of l-asparagine to l-aspartic acid, vanillylmandelic acid, and glutaric acid, yielding an AUC of 0.808.

### Autism spectrum disorders

In a study conducted by Chen et al., urine organic acids were detected in children with autism spectrum disorder (ASD) and combined with three algorithms, PLS-DA, SVM, and eXtreme Gradient Boosting (XGBoost), for the diagnosis of autism (Hung et al., 2021; Q. Chen et al., 2019). The work proved that autism spectrum disorders present with characteristic metabolic biomarkers that can be investigated for diagnosis of the condition as well as for future research on the pathogenesis of autism and possible interventions.

### Gestational age

Another application of ML in metabolomics is the investigation of the human pregnancy metabolome conducted by Liang et al. (Liang et al.,. 2020), where the authors were able to identify a series of compounds (460) and associated metabolic pathways (34) that were significantly changed during pregnancy. The authors were able to construct a linear regression model that correlates certain plasma metabolites with time in gestational age; this model is in high accordance with the ultrasound. An additional two to three metabolites were able to identify the time of labor, e.g., prediction of 2, 4, 6, or 8 weeks to the time of delivery.

## Methodological studies

The right choice of ML algorithm is a crucial factor for the success of a metabolomics study. Analysis results usually depend more on the data (type, quantity, quality) than the applied algorithm. Complex, multivariate approaches may be suitable for large, multidimensional datasets; however, in the case of simple, linearly separable data, conventional statistical approaches often outperform ML. Therefore, a large number of metabolomic studies make an effort to compare the predictive ability of different ML algorithms to each other, as well as to more traditional statistical methods.

One of the comprehensive comparative studies is the work by Mendez et al. (Mendez, Reinke, and Broadhurst 2019), in which the authors compared 8 ML algorithms, partial least squares regression (PLS), principal component regression (PCR), principal component logistic regression (PCLR), RF, linear kernel SVM, non-linear SVM with RBF, linear and non-linear ANN, for the binary classification of ten clinical metabolomic datasets. As for the ANNs, the linear network was composed of two layers, with a small number of linear neurons in the hidden layer and a single sigmoidal neuron in the output layer. For the non-linear NN, the activation function of the hidden layer neurons was changed to a sigmoidal function. Both networks were implemented using stochastic gradient descent with a binary cross-entropy loss function. The authors expected non-linear machine ML algorithms, especially the DL models, to outperform linear alternatives. Nevertheless, SVM and ANN only slightly surpassed PLS across all datasets, while RF performed poorly. In conclusion, no single DL or ML algorithm could be identified as superior.

In another 2019 study, Vu et al. evaluated the performance of five classification algorithms (PLS, OPLS, Principal component-Linear Discriminant Analysis (PC-LDA), RF, and SVM) using simulated and experimental 1D ^1^H NMR spectral data sets (Vu et al., 2019). Datasets with clear group separation were classified equally well by all five models. However, when the data contained subtle differences between classes, OPLS produced the best results, as it was able to identify the useful discriminant features with good classification accuracy. It is noteworthy that although RF and PC-LDA classified the data more accurately than the other models, they achieved so using the wrong discriminant features.

The superiority of SVM and RF classifiers was demonstrated in an evaluation of seven classification techniques using both simulated and real metabolomics datasets (Trainor, DeFilippis, and Rai 2017). In the simulated datasets, the classifiers performed as follows (from least to greatest error): SVM, RF, Naïve Bayes, sparse PLS, ANN, PLS, and *k*-NN, while SVM and RF consistently outperformed the rest over the real datasets.

Expanding on the gut microbiome, Han et al. (Han et al., 2021) used RF models to identify sets of metabolites that are able to provide taxonomic distinction and classify the origin of microbial supernatants while also providing insights into highly conserved chemical features that are predictive of taxonomic identity. Han et al. were able to construct a chemical standard library-informed metabolomics pipeline that is both customizable and expandable. This method was used to construct an atlas of metabolic activity that can enable functional studies of the gut microbial communities and was validated using RF ML algorithms.

## Concluding remarks

In this work, we provided a review of popular ML techniques as well as key studies that have applied them for the stratification of metabolites from various conditions.

RF and SVM have been among the most widely used algorithms in metabolomic studies. Although DL is a comparatively new player in the field, it is undoubtedly paving its way to metabolomics - and generally to the other omics and integrative multi-omics studies - as evident by the growing number of reports that use NNs in metabolomic analyses.

Cancer is by far the most studied condition, with ML algorithms having been applied to the supervised classification of cancer *versus* control sample sets from metabolic data obtained from various cancer types, including ovarian, breast, endometrial, lung and liver cancer, renal carcinoma, squamous cell carcinomas, osteosarcoma, and lymphomas.

Choosing the appropriate ML algorithm is crucial to the success of a metabolomics study. It is essential for researchers to be informed of the benefits of each ML approach and to choose one that best suits their needs to obtain reliable and interpretable outcomes. However, after reviewing a number of studies that compared different ML methods, no specific conclusion can be drawn regarding the choice of the algorithm. ML methods that produce good results in some investigations might perform poorly in others. The dimensionality, quality, and characteristics of input data and appropriate feature selection techniques play a significant role in the performance and behavior of the ML methods and their outcomes.

In addition, the choice of hyper-parameters and how they are tuned can influence the results remarkably. Accordingly, a detailed methodology for selecting the most suitable ML algorithm is a topic that needs further investigation. However, we can offer some insight into the pros and cons of each of the popular algorithms discussed in this review, as well as some suggested recommendations regarding their applications within the metabolomics field (Table 3) (Kell 2005; Kourou et al., 2015; Libbrecht and Stafford Noble, 2015; Soofi and Awan 2017; Malakar et al., 2018; Shinde and Shah 2018; Liebal et al., 2020).

**TABLE 3:** Pros and cons of ML algorithms and applicability within the field of metabolomics.

Algorithm	Pros	Cons	Metabolomic application
Linear Regression	- Excellent for linearly separable data	- Assumes linear relationship between dependent and independent variables	- Unknown relationship between dependent and independent variables
	- Easy implementation	- Outliers have significant impact	- Forecasting tasks
		- Prone to overfitting	
Logistic Regression	- Simple implementation	- Easily outperformed by other algorithms	- Multiclass classification, i.e., when output class only has two possible outcomes e.g., cancer detection (yes or no)
	- No Feature scaling needed	- Heavily reliant on proper identification of data	- Linear relationship between dependent and independent variables
	- No hyper-parameter tuning needed		
Naive Bayes	- Fast predictions of dataset classes	- Assumes all features are independent	- Dataset with highly independent features
	- Good for datasets with categorical variables		- For multi-class predictions
Support Vector Machines (SVMs)	- Works well for data that can be easily seperated with clear margin of separation	- Requires more training time for large datasets	- Medium sized dataset
	- Effective for high dimension data	- Does not perform well when dataset has high level of noise i.e. overlapping target classes	- Large number of features
			- Linear relationship between dependent and independent variables
*k*-Nearest Neighbors (*k*-NN)	- Easy implementation	- Slow performance on large datasets	- Small datasets with small number of features
	- Can solve multi-class problems	- Data scaling required	- Unknown relationship between dependant and independent variables
	- No data assumption needed	- Not for data with high dimensionality i.e. large number of features	- Useful for targeted metabolomics approaches
		- Sensitive to missing values, outliers and imbalance data	
Decision Trees	- Scaling or normalization of data not needed	- Data sensitive	- Known to suffer from a high chance of overfitting
	- Able to handle missing values	- Might need more time to train trees	
	- Easy to visualize	- High chance of overfitting	
	- Automatic feature selection		
Random Forest (RF)	- Good performance on imbalanced or missing data	- Predictions are uncorrelated	- Identification of variables with high importance
	- Able to handle huge amounts of data	- Influence of dependent variable on independent variable is unknown, i.e., Black box	- Useful for datasets with small sample population
	- Feature importance extraction	- Data sensitive	- Useful for metabolic fingerprinting approaches
	- Low chance of overfitting		
Neural Networks (NN)	- Flexible network architecture i.e., can be used for regression and classification	- Influence of dependent variable on the independent variable is unknown, i.e., Black box	- Data with a non-linear relationship between dependant and independent variables
	- Good with nonlinear data	- Highly dependant on training data	- Large datasets with a stipulation on time and cost
	- Can handle large number of inputs	- Prone to overfitting and generalization	- Can be applied to raw metabolomic data for feature extraction and multivariate classification combined into a single model
	- Fast predictions once trained	- Extremely hardware dependant i.e., the larger the datasets, the more expensive and time-consuming the modeling process	- Integration of multi-omics data, i.e., collected over different times, multiple analytical platforms, biofluids, or omic platforms
			- Useful for metabolic profiling

Significantly altered metabolites generated by metabolomic experiments and unveiled by machine learning approaches can serve as a starting point for a number of investigations. Biomarker discovery is a definite main target. Nevertheless, their actual predictive ability needs to be further experimentally validated. Further investigations like enrichment studies and pathway analysis can provide new insights into the roles the identified metabolites play in the pathophysiology of various conditions. Additionally, the feasibility of targeting specific metabolites for disease treatment can be explored.

It is noteworthy that most of the reviewed work was published within the last 5 years, which aligns with the obvious rise in popularity ML has gained in recent years, enabled by an increase in computation power, efficiency and accessibility of ML tools, familiarity with the field and abundance of data. As more and large metabolomic data sets become available, it is expected that ML techniques, especially DL, will play a bigger role in building informative and predictive models that can be used to provide high-definition, personalized clinical diagnosis, and treatment.

## Glossary

ML: Machine Learning
AI: Artificial Intelligence
DM: Diabetes mellitus
CVD: Cardiovascular Disease
NMR: Nuclear Magnetic Resonance
MS: Mass Spectrometry
AUC: Area Under the Curve
SVM: Support Vector Machine
*k*-NN: K-Nearest Neighbor
NN: Neural Networks
RF: Random Forests
UMAP: Uniform Manifold Approximation and Projection
t-SNE: t- stochastic neighbor embedding
PCA: Principle Component Analysis
ICA: Independent Component Analysis
PLS: Partial Least Squares
OPLS: Orthogonal Projections to Latent Structures
RBF: Radial Basis Function
DL: Deep Learning
ANN: Artificial Neural Network
CNN: Convolutional Neural Networks
PAM: Prediction Analysis for Microarrays
RPART: Recursive Partitioning And Regression Trees
PLS-DA: Partial Least Square-Discriminant Analysis
SDA: Stepwise discriminant Analysis
MLP: Multilayer perceptron
GTB: Gradient tree boosting
CKD: Chronic Kidney Disease
MDD: Major Depressive Disorders
XGBoost: eXtreme Gradient Boosting
PC-LDA: Principle component-Linear Discriminant Analysis
LASSO: Least Absolute Shrinkage and Selection Operator
PCR: Principal component regression
PCLR: Principal component logistic regression
