# Supplementary material for: Applications of machine learning in metabolomics: Disease modeling and classification
Source: Front Genet. 2022 Nov 24;13:1017340. doi: 10.3389/fgene.2022.1017340 (PMC9730048; doi:10.3389/fgene.2022.1017340)
Supplement: Supplementary file 1 [file Table1.docx]

**Supplementary Table 1. Metabolomic studies employing Support Vector Machine (SVM) algorithms**

| **Disease** | **Date of Publication** | **Metabolites Measured by** | **Feature Selection** | **Kernel Function** | **Results** | **Identified Metabolites** | **References** |
| --- | --- | --- | --- | --- | --- | --- | --- |
| **Cancer** | | | | | | | |
| Breast Cancer | 2009 | LC-ITMS  (Liquid chromatography ion trap mass spectrometry) | Oscillating Search Algorithm for Feature Selection (OSAF) | n/a | 83.5% sensitivity, 90.6%specifity  (10-fold cross validation)  83.5% sensitivity, 85.9 specificity  (Leave-one-out-cross-validation) | 44 identified pairwise metabolite combinations, dominated by  S-adenosylhomocysteine (SAH) | <https://doi.org/10.1186/1471-2407-9-104> |
| Breast Cancer | 2010 | GC-MS | Chi-square (univariate), Correlation-based method (multivariate), Decision tree and Random Forest (RF) (classifier-embedded methods) | Linear,  Gaussian RBF | > 90% overall accuracies  (10-fold cross-validation) | 5 metabolite panel, none identified | <https://doi.org/10.1186/1471-2105-11-S2-S4> |
| Hepatocellular Carcinoma | 2008 | GC-MS  (Gas chromatography/ mass spectrometry) | Stepwise discriminant analysis (SDA) | n/a | 75% accuracy  (20-fold cross validation) | 13 metabolite panel:  butanoic acid, ethanimidic acid, glycerol, L-isoleucine, L-valine, aminomalonic acid, D-erythrose, hexadecanoic acid, octadecanoic acid, 9,12-octadecadienoic acid, and three unidentified compounds | <https://doi.org/10.1002/rcm.3708> |
| Ovarian Cancer | 2005 | SELDI-TOF MS  (Surface-enhanced laser desorption and ionization time-of-flight mass spectrometry) | 4-step strategy dimensionality reduction: 1. binning, 2. Kolmogorov–Smirnov test, 3. restriction of coefficient of variation and 4. wavelet analysis | Gaussian RBF  (Gaussian radial basis function) | 97.38% average sensitivity, 93.30% average specificity  (1000 independent k-fold cross-validation) | n/a | <https://doi.org/10.1093/bioinformatics/bti370> |
| Ovarian Cancer | 2009 | LC-TOF MS  (Liquid chromatography time-of-flight mass spectrometry) | Recursive feature elimination (RFE), L1 SVM and Weston's feature selection method | Linear + degree 2 polynomial kernel | > 90% accuracy (leave-one-out-cross-validation, 12-fold-cross-validation, 52-20-split-validation) | n/a | <https://doi.org/10.1186/1471-2105-10-259> |
| Ovarian Cancer | 2015 | UPLC-MS  (Ultra-performance liquid chromatography mass spectrometry) | RFE | Linear | 100% accuracy  (Leave-one-out-cross-validation) | 16 metabolite panel, 11 Identified | <https://doi.org/10.1038/srep16351> |
| **Non-Cancer** | | | | | | | |
| Celiac Disease | 2009 | ^1^H NMR Spectroscopy | PLS | n/a | 79.7−83.4% accuracy for serum; 69.3% for urine | Serum: lower levels of amino acids, lipids, pyruvate, and choline  higher levels of glucose and 3-hydroxybutyric acid  Urine: altered levels of indoxyl sulfate, meta-[hydroxyphenyl]propionic acid and phenylacetylglycine. | <https://doi.org/10.1021/pr800548z> |
| Depression (Major Depressive Disorder with full remission) | 2021 | ^1^H NMR Spectroscopy | VIP of PLS-DA | Linear | ~ 85% accuracy | 8 metabolite panel:  histidine, succinic acid, proline, acetic acid, creatine, glutamine, glycine, and pyruvic acid, | <https://doi.org/10.1038/s41598-021-95221-1>  3 but nature |
| Multiple Sclerosis (beta interferon treatment resistance) | 2020 | NMR Spectroscopy | Homology reduction (removal of features with high correlation coefficients) | RBF | 84.2% and 82.2% accuracy in the two SVM models | n/a | <https://doi.org/10.3389/fimmu.2020.01527>  19 |
| Non-Alcoholic Steatohepatitis (NASH) and Liver Fibrosis | 2019 | MS, LC-MS, GC | RFE | Linear,  RBF | Up to 90% accuracy for differentiation between three conditions: non-alcoholic fatty liver disease, NASH, and liver fibrosis  98% accuracy for 10-lipid-model to detect liver fibrosis with | Combinations of glycans, lipids and hormones | <https://doi.org/10.1016/j.metabol.2019.154005>  73 |
